# The Exposure to Lead (Pb) Exacerbates Immunological Abnormalities in BTBR T^+^ Itpr^3tf^/J Mice through the Regulation of Signaling Pathways Relevant to T Cells

**DOI:** 10.3390/ijms242216218

**Published:** 2023-11-11

**Authors:** Mohammed A. Assiri, Thamer H. Albekairi, Mushtaq A. Ansari, Ahmed Nadeem, Sabry M. Attia, Saleh A. Bakheet, Mudassar Shahid, Abdullah A. Aldossari, Mohammed M. Almutairi, Taghreed N. Almanaa, Mohammad Y. Alwetaid, Sheikh F. Ahmad

**Affiliations:** 1 Department of Pharmacology and Toxicology, College of Pharmacy, King Saud University, Riyadh 11451, Saudi Arabiasbakheet@ksu.edu.sa (S.A.B.);; 2 Department of Pharmaceutics, College of Pharmacy, King Saud University, Riyadh 11451, Saudi Arabia; 3 Department of Botany and Microbiology, College of Science, King Saud University, Riyadh 11451, Saudi Arabiamywetaid@hotmail.com (M.Y.A.)

**Keywords:** autism spectrum disorder, lead (Pb), BTBR mice, inflammatory cytokines, regulatory T cells

## Abstract

Autism spectrum disorder (ASD) is a common neurodevelopmental illness characterized by abnormal social interactions, communication difficulties, and repetitive and limited behaviors or interests. The BTBR T^+^ Itpr3^tf^/J (BTBR) mice have been used extensively to research the ASD-like phenotype. Lead (Pb) is a hazardous chemical linked to organ damage in the human body. It is regarded as one of the most common metal exposure sources and has been connected to the development of neurological abnormalities. We used flow cytometry to investigate the molecular mechanism behind the effect of Pb exposure on subsets of CD4^+^ T cells in the spleen expressing IFN-γ, T-bet, STAT1, STAT4, IL-9, IRF4, IL-22, AhR, IL-10, and Foxp3. Furthermore, using RT-PCR, we studied the effect of Pb on the expression of numerous genes in brain tissue, including IFN-γ, T-bet, STAT1, STAT4, IL-9, IRF4, IL-22, AhR, IL-10, and Foxp3. Pb exposure increased the population of CD4^+^IFN-γ^+^, CD4^+^T-bet^+^, CD4^+^STAT1^+^, CD4^+^STAT4^+^, CD4^+^IL-9^+^, CD4^+^IRF4^+^, CD4^+^IL-22^+^, and CD4^+^AhR^+^ cells in BTBR mice. In contrast, CD4^+^IL-10^+^ and CD4^+^Foxp3^+^ cells were downregulated in the spleen cells of Pb-exposed BTBR mice compared to those treated with vehicle. Furthermore, Pb exposure led to a significant increase in IFN-γ, T-bet, STAT1, STAT4, IL-9, IRF4, IL-22, and AhR mRNA expression in BTBR mice. In contrast, IL-10 and Foxp3 mRNA expression was significantly lower in those treated with the vehicle. Our data suggest that Pb exposure exacerbates immunological dysfunctions associated with ASD. These data imply that Pb exposure may increase the risk of ASD.

## 1. Introduction

Autism spectrum disease (ASD) is a common neurodevelopmental condition characterized by poor social interaction, limited sociability, repetitive behavior, and stereotypical behavior [1]. Because of their high prevalence, high socioeconomic cost, and profound impact on families, this group of illnesses receives a lot of public attention. There is a lack of knowledge on the specific etiology of ASD. New evidence, however, suggests that immune dysfunction plays a significant role in the pathogenesis of ASD [2]. A recent study has shown that immune cells have a clear and undisputed role in the pathogenesis and development of ASD [3,4]. Previous research has shown a link between immunological alterations, including neuroinflammation and immune cell activation, and ASD [5,6]. Chemokine receptors are thought to have a role in various neuroinflammatory diseases [7]. Increased chemokine receptor levels have been associated with the emergence of behavioral problems in people with ASD [5,8]. Bakheet et al. [9] discovered that the disruption of signaling pathways connected with Th1, Th2, Th17, and T regulatory cells is linked to the severity of ASD development.

The immune system is essential in neurodevelopment and related processes [10]. Cytokines serve a crucial physiological role in brain development, cognitive functions, and behavior control [11,12]. Ahmad et al. [13] state that many cytokines, including IFN-γ, have been related to ASD. Previous research has shown an increase in the production of inflammatory mediators in the brain, cerebrospinal fluid, and serum of ASD patients [14,15]. Cao [16] discovered that people with ASD had significantly higher levels of IFN-γ in their plasma when compared to control subjects in experiments. According to Goines et al. [17], a raised level of IFN-γ is connected with an increased likelihood of conceiving a child with ASD. Filiano et al. [18] have shown that IFN-γ unexpectedly regulates brain connections and social behavior. The T-box transcription factor, T-bet, is crucial in differentiating Th1 cells [19]. T-bet expression is essential in the establishment of inflammatory reactions and their critical participation in autoimmune diseases [20]. T-bet expression is induced in B cells in humans [21]. Recent research by van Langelaar et al. [22] shows that T-bet-expressing cells are recruited into patients’ CNSs. There is compelling evidence that Th17 cells, proinflammatory T cells, and their cytokine IL-17A play a critical role in CNS disorders [23]. According to Orihuela et al. [24], the signaling pathway, including signal transducers and transcription 1 (STAT1) activators, is crucial in regulating microglial proinflammatory responses. STAT4 is classified as a member of the STAT family of transcription factors and functions as a transcriptional regulator specific to Th1 cells, as reported by Thierfelder et al. in [25]. This discovery highlights the importance of STAT4 in chronic CNS inflammation, which functions independently of the typical Th1 pathway [26,27].

Several earlier studies have shown that Th9 cells are responsible for the onset of neuroinflammation. A previous work carried out by Li et al. [28] indicated that IL-9 plays a significant role in T cell activation and the formation of autoimmune inflammation in the CNS. According to Mesples et al. [29], there is evidence that IL-9 is involved in brain cells. Kovac et al. [30] discovered a significant increase in IL-9 production in brain pericytes. According to the research performed by Ormstad et al. [31], there was an increase in IL-9 levels among those suffering from acute ischemia. IRF4 (interferon regulatory factor) is expressed in various immune cell types, including lymphocytes, macrophages, and dendritic cells. Th22 cells contribute significantly to IL-22, which is essential in multiple neurological diseases due to its potential to enhance leukocyte infiltration into the brain [32]. Wang et al. [33] found that IL-22 has a regulating function in the production of chemoattractants by microvascular endothelial cells in the blood–brain barrier (BBB). The aryl hydrocarbon receptor (AhR) has been linked to inflammatory reactions. Lee et al. [34] showed that AhR is responsible for mediating the inflammatory effects seen in microglia. Furthermore, Cuartero et al. [35] demonstrated that AhR is causal in the onset of brain damage.

Regulatory T cells (Treg cells) have long been recognized as critical players in the immune system. T helper and Treg cell dysregulation, as well as their related cytokines and transcription factors, may significantly influence the pathophysiology of neuroinflammatory disorders. According to the research, IL-10 is a very efficient anti-inflammatory cytokine that plays a vital role in the pathophysiology of ASD [36,37]. Previous research has shown that people with ASD have lower levels of Treg cells [38,39]. Treg cells serve an essential function in reducing immune activation and avoiding self-reactivity. According to Ahmad et al. [39] and Enstrom et al. [40], the absence or inadequacy of these cells has been associated with the development of autoimmune and neuroinflammatory disorders.

Lead (Pb) is a common environmental neurotoxicant with a variety of possible effects on children’s cognitive development. Pb is a dangerous neuro-developmental toxic chemical linked to neuroinflammatory processes in the brain [41]. Deficits in cognitive ability, memory, attention, and language are among the consequences [42,43,44]. According to Bleecker et al. [45], individuals exposed to Pb exhibited a significant loss in their capacity to recall words compared to those not acutely exposed. Furthermore, people exposed to Pb typically report deficiencies in their reading and language ability and decreases in IQ scores, social interaction, and memory [45,46,47]. Previous research has shown a link between higher Pb levels in plasma and cognitive skills and IQ scores [48]. Previous research has shown that most people exposed to Pb experience a deterioration in memory performance [46,47]. This discovery demonstrates that Pb exposure might impair memory and learning ability. Furthermore, this study suggests that Pb exposure may contribute to the development of memory impairment, a frequent co-occurring symptom in people with ASD. Previous study has shown that Pb exposure increases the number of macrophages and lymphocytes in the blood [49]. According to research by Lahat et al. [50], exposure to Pb causes an increase in proinflammatory cytokines within glial cells. Furthermore, Pb exposure has been linked to putative pro-inflammatory effects in the CNS [51]. This shows that Pb may have an impact on immunological systems in the brain, as well as other immune dysfunctions associated with the pathophysiology of ASD [52].

BTBR mice display classic ASD symptoms, such as repetitive behavior and decreased sociability [53,54]. Estes and McAllister [55] discovered comparable immunological alterations in ASD animal models, suggesting a link between abnormal CNS development and the presentation of ASD-like behaviors in offspring. Onore et al. [56] previously established that a changed immunological profile was involved in the social interaction of BTBR mice. In BTBR mice, Ahmad et al. [13] discovered that an elevated ratio of pro-inflammatory lymphocytes caused abnormal brain development, repetitive behaviors, and decreased social communication. The expression of proinflammatory mediators is altered in BTBR mice, which is linked to behavioral issues [57,58]. Previous research has shown a link between immunological issues and the appearance of an ASD behavioral pattern in BTBR mice [9,59,60]. Recent investigations by Ansari et al. [59] and Bakheet et al. [9] found the dysregulation of inflammatory cytokines and transcription factor signaling in BTBR mice and children with ASD. According to our previous investigation, Pb exposure increased the number of marbles buried by mice and substantially increased their repeated behavior. Furthermore, the research found that Pb exposure negatively influenced social behavior [61]. The present study examined the impact of Pb exposure on several inflammatory pathways in BTBR mice.

## 2. Results

### 2.1. The Administration of Pb Results in the Upregulation of Transcription Factors Associated with Th1 Cells in BTBR Mice

**Figure 1:**
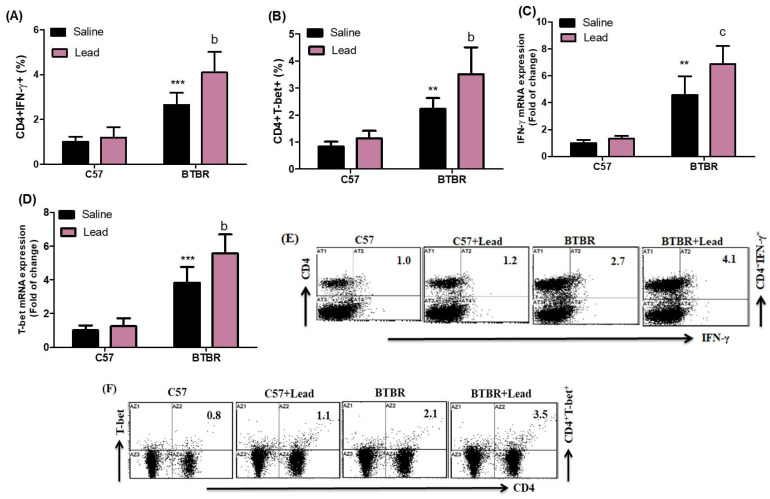
(**A**,**B**) The impact of Pb exposure on the population of CD4^+^ T lymphocytes expressing IFN-γ and T-bet in the spleen cells. (**C**,**D**) The IFN-γ and T-bet mRNA expression levels in brain tissue were analyzed using RT-PCR. (**E**,**F**) The dot plots representing CD4^+^IFN-γ^+^ and CD4^+^T-bet^+^ cells were acquired using flow cytometry for each sample of spleen cells. BTBR and C57 mice were subjected to oral administration of Pb at a dosage of 0.1 mg/kg/day for eight weeks. The C57 and BTBR mice were administered saline orally. The statistical analysis was conducted using a two-way analysis of variance (ANOVA) followed by Tukey’s post hoc test. The data are presented as mean ± standard deviation (n = 6 in each group). * *p* < 0.05, ** *p* < 0.01, and *** *p* < 0.001 compared with saline-treated C57 mice; ^a^ *p* < 0.05, ^b^ *p* < 0.01, and ^c^ *p* < 0.001 compared with saline-treated BTBR mice.

This work aims to look into the effect of Pb exposure on the signaling of Th1 cell-related transcription factors in C57 and BTBR mice. In this study, the quantification of IFN-γ and T-bet-expressing CD4^+^ T cells was conducted within the spleen. The treatment of BTBR mice with Pb resulted in a significant increase in the expression of IFN-γ and T-bet in CD4^+^ T cells compared to BTBR mice treated with the vehicle. This increase was observed for the strain effect (F _(1,20)_ = 90.59, *p* = 0.0001), exposure effect (F _(1,20)_ = 11.78, *p* = 0.0026), and exposure × strain effect (F _(1,20)_ = 6.890, *p* = 0.00162) in CD4^+^IFN-γ^+^ cells, and strain effect (F_(1,20)_ = 67.91, *p* = 0.0001), exposure effect, (F_(1,20)_ = 12.24, *p* = 0.0023), and exposure × strain effect (F_(1,20)_ = 4.635, *p* = 0.0437) in CD4^+^T-bet^+^ cells (Figure 1A,B). To obtain a more thorough understanding of the molecular mechanism of Pb exposure, we performed an RT-PCR investigation to determine changes in the gene expression of IFN-γ and T-bet in brain tissue. Our findings show that BTBR mice treated with Pb had greater levels of IFN-γ and T-bet mRNA than BTBR mice treated with a vehicle. This effect was observed for both the strain effect (F_(1,20)_ = 131.8, *p* = 0.0001), exposure effect (F _(1,20)_ = 55.49, *p* = 0.0001), and exposure × strain effect (F _(1,20)_ = 50.01, *p* = 0.0001) for IFN-γ mRNA (Figure 1C) and the strain effect (F_(1,20)_ = 125.7, *p* = 0.0001), exposure effect (F_(1,20)_ = 9.639, *p* = 0.0056), and exposure × strain effect (F_(1,20)_ = 5.456, *p* = 0.0300) for T-bet mRNA (Figure 1D). As a result, our data suggest that Pb exposure may cause immunological abnormalities in BTBR mice via increasing the expression of Th1. 

### 2.2. Exposure to Pb Resulted in an Upregulation of STAT1 and STAT4 Expression in BTBR Mice

**Figure 2:**
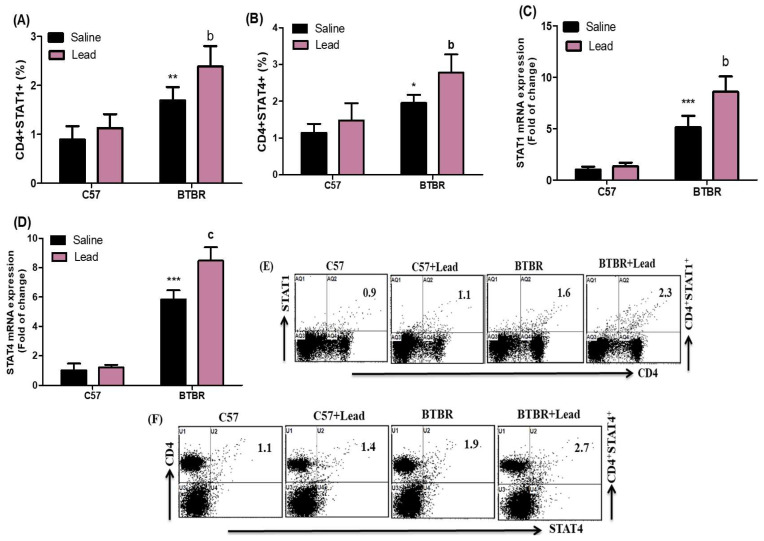
(**A**,**B**) The impact of Pb exposure on the population of CD4^+^ T lymphocytes expressing STAT1 and STAT4 in the spleen cells. (**C**,**D**) Analyzed STAT1 and STAT4 mRNA expression levels in brain tissue via RT-PCR. (**E**,**F**) The dot plots representing CD4^+^STAT1^+^ and CD4^+^STAT4^+^ cells were acquired using flow cytometry for each sample of spleen cells. The statistical analysis was conducted using a two-way analysis of variance (ANOVA) followed by Tukey’s post hoc test. The data are presented as mean ± standard deviation (n = 6 in each group). * *p* < 0.05, ** *p* < 0.01, and *** *p* < 0.001 compared with saline-treated C57 mice; ^a^ *p* < 0.05, ^b^ *p* < 0.01, and ^c^ *p* < 0.001 compared with saline-treated BTBR mice.

Further research is needed to perform a more detailed evaluation of the influence of Pb on the proportion of CD4^+^ T cells expressing STAT1 and STAT4 in the spleen. The findings of our investigation show that the proportion of CD4^+^ T cells expressing STAT1 and STAT4 is much higher in Pb-treated BTBR mice than in vehicle-treated BTBR mice. This increase was observed for both CD4^+^STAT1^+^ cells for the strain effect (F_(1,20)_ = 62.39, *p* = 0.0001), exposure effect (F_(1,20)_ = 12.58, *p* = 0.0020), and exposure × strain effect (F_(1,20)_ = 2.987, *p* = 0.0076) in the CD4^+^STAT1^+^ cells and for the strain effect (F_(1,20)_ = 62.39, *p* = 0.0001), exposure effect, (F_(1,20)_ = 12.58, *p* = 0.0020), and exposure × strain effect (F_(1,20)_ = 2.987, *p* = 0.0453) in the CD4^+^STAT4^+^ cells (Figure 2A,B). To provide a more comprehensive elucidation of the impact of Pb on the genetic expression of STAT1 and STAT4 inside brain tissue, the results of our study indicate that BTBR mice treated with Pb exhibited higher levels of STAT1 and STAT4 mRNA than BTBR mice treated with a vehicle. This difference was observed for the strain effect (F_(1,20)_ = 212.1, *p* = 0.0001), exposure effect (F_(1,20)_ = 23.13, *p* = 0.0001), and exposure × strain effect (F_(1,20)_ = 15.76, *p* = 0.0008) in the STAT1 mRNA (Figure 2C). The statistical analysis revealed significant differences in the strain effect (F_(1,20)_ = 603.8, *p* = 0.0001), exposure effect (F_(1,20)_ = 32.70, *p* = 0.0001), and exposure × strain effect (F_(1,20)_ = 25.13, *p* = 0.0001) in the STAT4 mRNA, as depicted in Figure 2D. The findings of our study indicate that exposure to Pb increased the signaling of transcription factors related to Th1 cells. This increase in signaling might lead to neuroimmune dysfunction in BTBR animals. 

### 2.3. Exposure to Pb Has Been Found to Enhance Th9 Signaling in BTBR Mice

**Figure 3:**
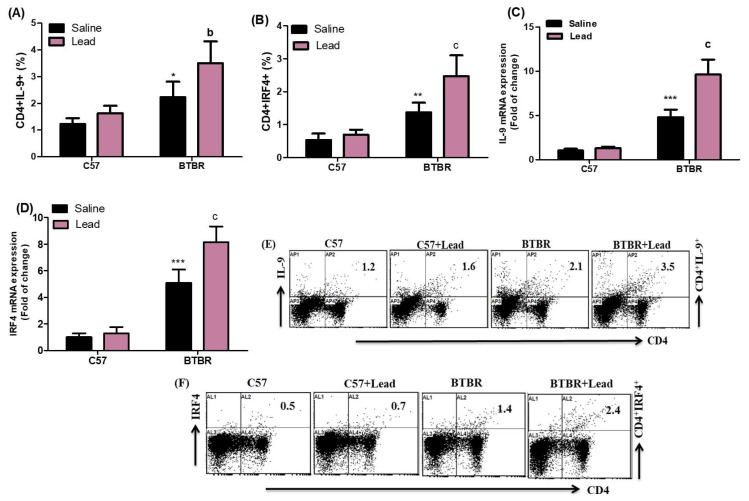
(**A**,**B**) The impact of Pb exposure on the population of CD4^+^ T lymphocytes expressing IL-9 and IRF4 in the spleen cells. (**C**,**D**) The analysis of IL-9 and IRF4 mRNA expression levels in brain tissue was conducted using RT-PCR. (**E**,**F**) The dot plots representing CD4^+^IL-9^+^ and CD4^+^IRF4^+^ cells were acquired using flow cytometry for each sample of spleen cells. The statistical analysis was conducted using a two-way analysis of variance (ANOVA) followed by Tukey’s post hoc test. The data are presented as mean ± standard deviation (n = 6 in each group). * *p* < 0.05, ** *p* < 0.01, and *** *p* < 0.001 compared with saline-treated C57 mice; ^a^ *p* < 0.05, ^b^ *p* < 0.01, and ^c^ *p* < 0.001 compared with saline-treated BTBR mice.

Our research findings show a considerable increase in the number of CD4^+^ T cells expressing IL-9 and IRF4 in the spleen cells of BTBR mice treated with Pb vs. BTBR mice treated with a vehicle. This increase was observed for both the strain effect (F_(1,20)_ = 43.94, *p* = 0.0001), exposure effect (F_(1,20)_ = 14.93, *p* = 0.0010), and exposure × strain effect (F_(1,20)_ = 3.981, *p* = 0.0458) of CD4^+^IL-9^+^ cells and for the strain effect (F_(1,20)_ = 108.1, *p* = 0.0001), exposure effect (F_(1,20)_ = 14.28, *p* = 0.0012), and exposure × strain effect (F_(1,20)_ = 7.993, *p* = 0.0107) of CD4^+^IRF4^+^ cells (Figure 3A,B). The mRNA levels of IL-9 and IRF4 were evaluated in the brain tissue. IL-9 and IRF4 mRNA expression levels in the brain tissue significantly increased in Pb-exposed BTBR mice compared to vehicle-treated BTBR mice. This increase was observed for both the strain effect (F_(1,20)_ = 244.2, *p* = 0.0001), exposure effect (F_(1,20)_ = 43.42, *p* = 0.0001), and exposure × strain effect (F_(1,20)_ = 34.57, *p* = 0.0001) in the case of IL-9 mRNA expression levels, and for the strain effect (F_(1,20)_ = 226.9, *p* = 0.0001), exposure effect (F_(1,20)_ = 24.39, *p* = 0.0001), and exposure × strain effect (F_(1,20)_ = 16.99, *p* = 0.0008) in the case of IRF4 mRNA expression levels (Figure 3C,D). The data reported here show that Pb exposure causes Th9 cell upregulation in BTBR mice. 

### 2.4. Exposure to Pb Induces an Upregulation of the Transcription Factor Associated with the Th22 Immune Response in BTBR Mice

**Figure 4:**
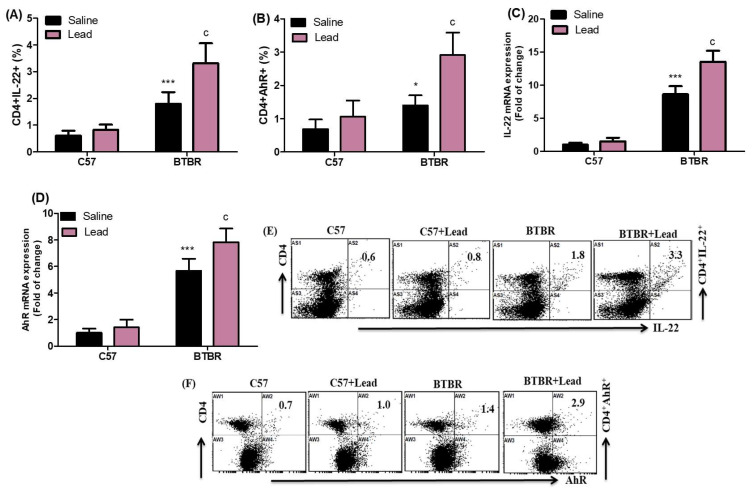
(**A**,**B**) The impact of Pb exposure on the population of CD4^+^ T lymphocytes expressing IL-22 and AhR in the spleen cells. (**C**,**D**) The analysis of IL-22 and AhR mRNA expression levels in brain tissue was conducted using RT-PCR. (**E**,**F**) The dot plots representing CD4^+^IL-22^+^ and CD4^+^AhR^+^ cells were acquired using flow cytometry for each sample of spleen cells. The statistical analysis was conducted using a two-way analysis of variance (ANOVA) followed by Tukey’s post hoc test. The data are presented as mean ± standard deviation (n = 6 in each group). * *p* < 0.05, ** *p* < 0.01, and *** *p* < 0.001 compared with saline-treated C57 mice; ^a^ *p* < 0.05, ^b^ *p* < 0.01, and ^c^ *p* < 0.001 compared with saline-treated BTBR mice.

Further experiments were conducted to perform a more thorough investigation of the effect of Pb on the proportion of IL-22- and AhR-expressing CD4^+^ T cells in the spleen cell population. Pb-exposed BTBR mice had a more significant percentage of CD4^+^ T cells expressing IL-22 and AhR than vehicle-treated BTBR mice. This difference was significant for the strain effect (F _(1,20)_ = 99.21, *p* = 0.0001), exposure effect (F _(1,20)_ = 22.06, *p* = 0.0001), and exposure × strain effect (F _(1,20)_ = 12.30, *p* = 0.0022) in the CD4^+^IL-22^+^ cells. Similarly, for CD4^+^AhR^+^ cells, the difference was significant for the strain effect (F (1,20) = 45.64, *p* = 0.0001), exposure effect (F (1,20) = 24.63, *p* = 0.0001), and exposure × strain effect (F (1,20) = 9.171, *p* = 0.0066) (Figure 4A,B). To further our understanding of the impact of Pb exposure, an investigation was conducted to assess the levels of IL-22 and AhR mRNA in brain tissue. Pb exposure in BTBR mice significantly upregulates IL-22 and AhR mRNA expression levels compared with those of vehicle-treated BTBR mice for the strain effect (F_(1,20)_ = 508.6, *p* = 0.00001), exposure effect (F_(1,20)_ = 38.73, *p* = 0.0001), and exposure × strain effect (F_(1,20)_ = 25.50, *p* = 0.0001) in the IL-22 mRNA expression levels and for the strain effect (F_(1,20)_ = 316.2, *p* = 0.0001), exposure effect (F_(1,20)_ = 17.00, *p* = 0.0005), and exposure × strain effect (F_(1,20)_ = 7.996, *p* = 0.0104) in the AhR mRNA expression levels (Figure 4C,D). The findings of our study demonstrated that exposure to Pb resulted in an upregulation of Th22 cells in BTBR mice.

### 2.5. The Exposure to Pb Results in a Reduction in the Transcription Factor Associated with T Reg Cells in BTBR Mice

**Figure 5:**
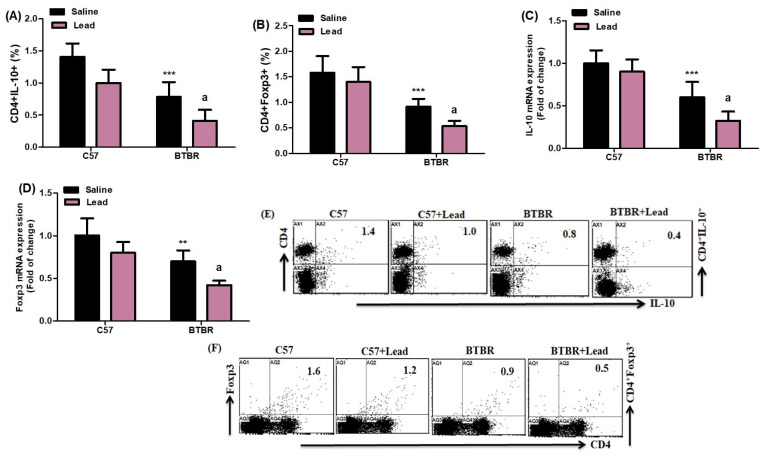
(**A**,**B**) The impact of Pb exposure on the population of CD4^+^ T lymphocytes expressing IL-10 and Foxp3 in the spleen cells. (**C**,**D**) The analysis of IL-10 and Foxp3 mRNA expression levels in brain tissue was conducted using RT-PCR. (**E**,**F**) The dot plots representing CD4^+^IL-10^+^ and CD4^+^Foxp3^+^ cells were acquired using flow cytometry for each sample of spleen cells. The statistical analysis was conducted using a two-way analysis of variance (ANOVA) followed by Tukey’s post hoc test. The data are presented as mean ± standard deviation (n = 6 in each group). * *p* < 0.05, ** *p* < 0.01, and *** *p* < 0.001 compared with saline-treated C57 mice; ^a^ *p* < 0.05, ^b^ *p* < 0.01, and ^c^ *p* < 0.001 compared with saline-treated BTBR mice.

The present study aimed to comprehensively assess the impact of Pb exposure on the proportion of CD4^+^ T cells expressing IL-10 and Foxp3 in spleen cells. A decrease in the percentage of IL-10- and Foxp3-expressing CD4^+^ T cells was observed in Pb-exposed BTBR mice compared to vehicle-treated BTBR mice. The current work sought to investigate the effect of Pb exposure on the fraction of CD4+ T cells expressing IL-10 and Foxp3 in spleen cells. Pb-exposed BTBR mice had fewer IL-10- and Foxp3-expressing CD4^+^ T cells than vehicle-treated BTBR mice. This decrease was significant for the strain effect (F_(1,20)_ = 51.36, *p* = 0.0001), exposure effect, (F_(1,20)_ = 21.23, *p* = 0.0002), and exposure × strain effect (F_(1,20)_ = 3.041, *p* = 0.0343) in the CD4^+^IL-10^+^ cells. Similarly, a significant decrease was observed for the strain effect (F_(1,20)_ = 62.85, *p* = 0.0001), exposure effect (F_(1,20)_ = 8.590, *p* = 0.0083), and exposure × strain effect (F_(1,20)_ = 1.094, *p* = 0.0451) in the CD4^+^Foxp3^+^ cells (Figure 5A,B). Subsequently, the mRNA expression levels of IL-10 and Foxp3 were evaluated in the brain tissue. The results of our study indicate that exposure to Pb led to a decrease in the expression of IL-10 and Foxp3 mRNA compared to the control mice of the BTBR strain. This decrease was observed for the strain effect (F_(1,20)_ = 65.57, *p* = 0.0001), exposure effect, (F_(1,20)_ = 9.753, *p* = 0.0050), and exposure × strain effect (F_(1,20)_ = 2.146, *p* = 0.0376) in the IL-10 mRNA expression level. Similarly, for the Foxp3 mRNA expression level, the decrease was observed for the strain effect (F_(1,20)_ = 37.73, *p* = 0.0001), exposure effect (F_(1,20)_ = 18.88, *p* = 0.0003), and exposure × strain effect (F_(1,20)_ = 2.196, *p* = 0.0157) (Figure 5C,D). The findings of our study indicate that exposure to Pb is associated with immunological dysregulation, potentially exacerbating the neuroinflammatory processes implicated in neurological and autism diseases. 

## 3. Discussion

Prior research found that high Pb exposure may affect children’s intellect, including deficiencies in cognitive function, attention, and memory [43,62]. Previous research has also shown abnormalities in several brain functions after Pb exposure, as demonstrated by Mason et al. [43], Kim and Kang [63], and Radulescu and Lundgren [64]. Pb has been connected to neurotransmitter release and reuptake regulation [65]. In general, alterations in the cholinergic, dopaminergic, serotonergic, and glutamatergic systems are frequently observed in people exposed to Pb, as reported by Akinyemi et al. [65], Mason et al. [43], Kim and Kang [63], and Radulescu and Lundgren [64]. Pb is a significant environmental contaminant, and extensive research has substantiated its detrimental effects on human health. The presence of Pb in the human body can have diverse effects on the CNS [66,67]. According to a study conducted by Sobin et al. [68], their prior investigation examined the impact of early chronic Pb exposure on microglial dysfunction in young mice. In [69], Canfield et al. conducted research on the effects of low-level Pb exposure on the hippocampus. Previous research has shown that Pb exposure during the neonatal period alters the hippocampus’s structure, cellular components, and genetic properties [70]. Furthermore, earlier research has shown that Pb exposure during the neonatal period enhances the phosphorylation of Tau protein in both the cerebral cortex and the cerebellum [71].

The expression levels of IFN-γ were increased in children diagnosed with ASD [72]. Previous research conducted by Li et al. [14] has demonstrated an elevation in the levels of IFN-γ in the brains of autistic patients. Another previous study reported an elevation in the IFN-γ level in autistic children [73]. Furthermore, it has been shown that mothers of children with ASD had an increase in IFN-γ cytokine levels [17]. A previous work by Nadeem et al. [74] demonstrated the relevance of the link between IFN-γ levels in ASD patients and the inflammatory process associated with ASD. This process occurs via a mechanism independent of T-bet, as noted by Grifka-Walk and Segal [75]. The protein STAT4 is essential in properly functioning innate and adaptive immune cells; Mathur et al. [76] found that its principal function is to promote the growth of Th1 and Th17 cells. The STAT4 gene has been related to an increased vulnerability to autoimmune illnesses such as SLE, primary Gren’s syndrome, rheumatoid arthritis, and thyroid problems [77,78]. Several genetic association studies have shown evidence that mutations in the STAT4 gene are connected to an increased vulnerability to various autoimmune diseases [77,79,80]. The purpose of this research was to look at the effect of Pb exposure on Th1 cell levels in BTBR mice. Following Pb exposure, there was an increase in the number of CD4^+^ T cells expressing IFN-γ, T-bet, STAT1, and STAT4 in the spleen of BTBR mice. We performed a more in-depth analysis of the effects of Pb exposure on BTBR mice in this work. We specifically looked at the mRNA expression levels for IFN-γ, T-bet, STAT1, and STAT4 in the brain tissue of Pb-exposed BTBR mice. Our results demonstrated that the expression levels of these genes increased significantly in Pb-exposed BTBR mice. According to this research, the presence of Pb alters Th1 cells, perhaps leading to an increase in inflammatory responses. Our data imply that Pb exposure may increase Th1 expression and contribute to the worsening of ASD development. As a result, the results of this research suggest that Pb exposure may increase the chance of ASD development.

IL-9 has been identified as a pro-inflammatory factor involved in developing autoimmune and neuroinflammatory illnesses [81]. Recent research has identified IL-9 as a distinct subpopulation of Th9 cells connected to various inflammatory diseases [82]. There have been reports of IL-9 activation in children with ASD, revealing a possible relationship between IL-9 and the neurodevelopmental elements of ASD, according to Alzghoul et al. [83]. When exposed to inflammatory stimuli, IL-9 signaling affects the cells that reside in the CNS. Furthermore, Ding et al. [84] and Saresella et al. [85] found an increase in IL-9 levels in neurodegenerative disorders. According to Zhou et al. [86], the cytokine IL-9 has been identified as a pro-inflammatory factor in neuroinflammatory disorders’ etiology. Previous research has shown that both IL-9 and its associated receptor are expressed in the CNS [87]. Previous research presented the first evidence that IL-9 has a role in CNS inflammation [28]. Recent research has demonstrated a significant increase in the amount of IL-9 in children with ASD and the BTBR autistic mouse model [88,89]. This data suggests that IL-9 may have a role in the development of ASD. An increase in IL-9 levels was seen in prior research conducted by Ahmad et al. [88] on children diagnosed with ASD. This research suggests a link between IL-9 and the neurological processes associated with ASD. Our findings show a significant increase in the number of IL-9- and IRF4-expressing CD4^+^ T cells in the spleens of BTBR mice after Pb treatment. The current work adds to the data that IL-9 and IRF4 mRNA expression levels are increased in the brains of Pb-treated BTBR mice. Pb exposure resulted in a significant increase in Th9 cells. Our data suggest that Pb exposure may cause immunological problems in BTBR mice by causing an increase in Th9 cells. The findings of this research indicate that the presence of Pb in BTBR mice may have a role in the development of ASD.

Previous research has indicated the presence of IL-22 in various clinical diseases, with a higher expression associated with lymphocyte activation in the CNS [88]. The cytokine IL-22 is essential in beginning an inflammatory response and so contributes to the inflammation seen in neurodevelopmental disorders [89]. Previous research has shown that IL-22 plays a critical role in human diseases and that its overexpression is connected with brain-activated lymphocytes [90]. According to research by Kebir et al. [91], the increased expression of IL-22 enhances leukocyte infiltration into the brain. In contrast, Elyaman et al. [92] discovered a link between the overexpression of the cytokine IL-22 and the onset of neurodegenerative diseases. Several investigations have shown that IL-22 overexpression is associated with immunological impairment in children with ASD and BTBR mice [93]. The results of this research provide empirical support for the idea that Th22 signaling may be an essential biomarker for immunological dysregulation in people with ASD. We discovered the chemical mechanism behind the effect of Pb exposure on Th22 cells in our work. Our data show a considerable increase in IL-22- and IRF4-expressing CD4^+^ T cells in the spleens of Pb-exposed BTBR mice. To better understand how Pb exposure affects brain tissues, we found that the mRNA expression levels of IL-22 and IRF4 in BTBR mice increased significantly when exposed to Pb. Following Pb exposure, the expression of Th22 was increased dramatically in both spleen and brain tissue, according to this research. Our data suggest that Pb exposure may significantly influence the development of immunological dysfunction in BTBR mice.

According to research by Lu et al. [94] and Vahedi et al. [95], Treg cells have an essential role in avoiding immune-mediated inflammatory diseases. Treg cells have an essential role in immune system modulation and the prevention of immunopathological disorders, as proven by Yamano et al. [96] and Yu et al. [97]. Several recent research investigations have looked into the loss of Treg cells in the brain tissues, peripheral blood, and spleen cells of ASD animal models and children [9,39]. Treg cells maintain self-tolerance by releasing the anti-inflammatory cytokine IL-10 [98,99]. Numerous studies have been carried out to evaluate the role of IL-10 as a well-established immunosuppressive cytokine, and its relationship with Treg cell activities has been well investigated [2,20]. Previous study has shown that BTBR mouse models had lower levels of IL-10, indicating an exacerbated state of inflammation [56,100]. According to our results, there was a substantial decrease in the number of CD4^+^IL-10^+^ and CD4^+^Foxp3^+^ cells, as well as the expression of IL-10 and Foxp3 mRNA, in the spleen and brain tissue of BTBR mice after Pb exposure. Pb’s ability to downregulate Treg cells accounts for the observed pro-inflammatory effect in BTBR animals. As a result, the results of this research indicate that Pb might lower the number of Treg cells, perhaps contributing to the development of autism-like symptoms in BTBR mice.

The findings presented in this study indicate that exposure to Pb is associated with an upregulation of proinflammatory markers. The results presented in this study support the hypothesis that exposure to Pb significantly diminishes anti-inflammatory signals, highlighting a potentially significant association between Pb and ASD. The findings of this research indicate that exposure to Pb may potentially have a role in the onset of neurobehavioral and immunological dysfunctions that are often associated with ASD.

## 4. Materials and Methods

### 4.1. Chemical and Reagents

Lead (Pb), PMA, ionomycin, and RPMI medium were purchased from Sigma-Aldrich (St. Louis, MI, USA). The Fc receptor (FcR) blocking reagent was purchased from Miltenyi Biotech (Bergisch Gladbach, Germany). The PE-anti-CD4, FITC-anti-CD4, PE/Dazzle-anti-CD4, APC/Cy7-anti-CD4, PE-anti-IFN-γ, PE/Dazzle-anti-T-bet, FITC-ant-STAT1, APC/Cy7-anti-STAT4, PE-anti-IL-9, FITC-anti-IRF4, PE-anti-IL-22, APC-anti-AhR, PE-anti-IL-10, PE/Dazzle-anti-Foxp3, and permeabilization and fixation buffers were purchased from BioLegend (San Diego, CA, USA). The GolgiStop and RBC’s lysing buffer were purchased from BD Biosciences (San Diego, CA, USA). The primers were purchased from Genscript Piscataway (Piscataway, NJ, USA). SYBR^®^ Green PCR master mix and high-capacity cDNA reverse transcription kits were purchased from Applied Biosystems (Foster City, CA, USA). The TRIzol reagent was purchased from Life Technologies (Paisley, UK).

### 4.2. Animals and Experimental Design

Male mice of the C57BL/6 (C57) and BTBR T^+^ Itpr^3tf/^J (BTBR) strains, aged 8–10 weeks and weighing 25–30 g, were purchased from the Jackson Laboratory (Bar Harbour, ME, USA). The mice were housed in our animal facilities and subjected to a 12 h light and dark cycle. The temperature in the facilities was maintained at 22 ± 2 °C, and the mice were kept in a controlled environment free from specific pathogens. They had unrestricted access to water. The Institutional Animal Care and Use Committee, King Saud University, Riyadh, Saudi Arabia, approved all experimental methods. This study aimed to examine the impact of Pb exposure on C57 and BTBR mice. The mice were allocated into four distinct experimental groups, each comprising six animals. The control groups consisted of C57 and BTBR mice given water devoid of Pb. The second group consisted of C57 and BTBR mice, administered Pb at a concentration of 0.1 mg/L in their drinking water for eight weeks. The findings of prior investigations conducted by Almutairi et al. [61] and Luo et al. [101] informed the selection of the Pb dosage. Upon completion of the experiment, all mice were euthanized, and the spleen was then extracted to conduct flow cytometry analysis. The brain samples were preserved in liquid nitrogen, followed by storage at a temperature of −80 °C. This was carried out to facilitate further investigation of mRNA and protein expression.

### 4.3. Flow Cytometric Analysis

Flow cytometric analysis assessed the IFN-γ, T-bet, STAT1, STAT4, IL-9, IRF4, IL-22, AhR, IL-10, and Foxp3 expression in CD4 cells. In brief, splenocytes were incubated with PMA/ionomycin (Sigma-Aldrich) and Golgi-plug (BD Biosciences) for four hours [102]. Cells were then washed and surface stained using PE-anti-CD4, FITC-anti-CD4, PE/Dazzle-anti-CD4, and APC/Cy7-anti-CD4 (BioLegend). After fixation and permeabilization (BioLegend), the cells were stained using PE-anti-IFN-γ, PE/Dazzle-anti-T-bet, FITC-ant-STAT1, APC/Cy7-anti-STAT4, PE-anti-IL-9, FITC-anti-IRF4, PE-anti-IL-22, APC-anti-AhR, PE-anti-IL-10, and PE/Dazzle-anti-Foxp3 (BioLegend). The number of CD4^+^IFN-γ^+^, CD4^+^T-bet^+^, CD4^+^STAT1^+^, CD4^+^STAT4^+^, CD4^+^IL-9^+^, CD45^+^IRF4^+^, CD4^+^IL-22^+^, CD45^+^AhR^+^, CD4^+^IL-10^+^, and CD4^+^Foxp3^+^ cells were detected using flow cytometry and analyzed using FC500 CXP software (Beckman Coulter, Indianapolis, IN, USA).

### 4.4. RT-PCR Analysis

The manufacturer’s instructions were used to isolate RNA from the mouse brain using TRIzol reagent (Life Technologies, Paisley, UK). The cDNA was synthesized using a high-capacity reverse transcription kit manufactured by Applied Biosystems (Foster City, CA, USA). The primers utilized in this study were chosen from the NCBI database.: GAPDH, F: 5′-GGCAAATTCAACGGCACAGT-3′ and R: 5′-TGAAGTCGCAGGAGACAACC-3′; IFN-γ, F: 5′-TCTGGGCTTCTCCTCCTGCGG-3′ and R: 5′-GGCGCTGGACCTGTGGGTTG-3′; T-bet, F: 5′-TCAACCAGCACCAGACAGAG-3′ and R: 5′-AACATCCTGTAATGGCTTGTG-3′; STAT1, F: 5′-TGGGCGTCTATCCTGTGGTA-3′ and R: 5′-TGAATGTGATGGCCCCTTCC-3′; STAT4, F: 5′-AATCCGGCATCTGCTAGCTC-3′ and R: 5′-AGGTCCCTGGATAGGCATGT-3′; IL-9, F: 5′-ACCAGCTGCTTGTGTCTCTC-3′ and R: 5′-CGGCTTTTCTGCCTTTGCAT-3′; IRF4, F: 5′- GGGTGCTTTCTGTTGGCTTG-3′ and R: 5′-CTGGCTTGCCAAACACTGTC-3′; IL-22, forward: 5′-TGCAAGCTTGAGGTGTCCAA-3′ and reverse: 5′-GAAGGCAGGAAGGAGCAGTT-3′; AhR, F: 5′-TTCAGAACTGACTCCACCGC-3′ and R: 5′-CCGGGTGTGATATCGGGAAG-3′; IL-10, F: 5′-ACCTGCTCCACTGCCTTGCT-3′ and R: 5′-GGTTGCCAAGCCTTATCGGA-3′; Foxp3, F: 5′-GGTATATGCTCCCGGCAACT-3′ and R: 5′-GATCATGGCTGGGTTGTC-3′. Relative fold changes in gene expression were determined using the 2^−ΔΔCT^ method [103] with *GAPDH* as the reference gene.

### 4.5. Statistical Analysis

The data are reported as the mean ± standard deviation (n = 6). The data were subjected to a two-way analysis of variance (ANOVA); subsequently, Bonferroni’s post hoc test was employed to conduct multiple comparisons. Statistical analysis was performed using GraphPad Prism 5.0. A significance level of *p* < 0.05 was deemed to indicate statistical significance.

## Data Availability

All data presented in this study are available on reasonable request from the corresponding author.

## Abbreviations

Lead (Pb): central nervous system (CNS); autism spectrum disorder (ASD); cluster of differentiation (CD); C57BL/6 (C57); BTBR T^+^ Itpr3^tf^/J (BTBR); Blood–brain barrier (BBB); Phorbol 12-myristate 13-acetate (PMA); Phycoerythrin (PE); Fluoroisothiocyanate (FITC); allophycocyanin (APC); messenger RNA (mRNA); reverse transcription polymerase chain reaction (RT-PCR); complementary DNA (cDNA); Glyceraldehyde 3-phosphate dehydrogenase (GAPDH); Roswell Park Memorial Institute (RPMI) 1640; Anti-Aryl hydrocarbon Receptor (AhR); Forkhead box protein 3 (FoxP3); red blood cell (RBC); Signal Transducer And Activator Of Transcription 1 (STAT); Interferon gamma (IFN-γ); Interferon Regulatory Factor 4 (IRF4); T-box gene expressed in T cells (T-bet); Regulatory T cells (Treg cells).
